# Synthesis and Characterization of ZnO and TiO_2_ Hybrid Coatings for Textile UV Anti-Aging Protection

**DOI:** 10.3390/polym16142001

**Published:** 2024-07-12

**Authors:** Maja Somogyi Škoc, Jelena Macan, Suzana Jakovljević, Iva Rezić

**Affiliations:** 1 Department of Materials, Fibres and Textile Testing, Faculty of Textile Technology, University of Zagreb, Prilaz baruna Filipovića 28a, HR-10000 Zagreb, Croatia; 2 Faculty of Chemical Engineering and Technology, University of Zagreb, Marulićev trg 19, HR-10001 Zagreb, Croatia; jmacan@fkit.unizg.hr; 3 Faculty of Mechanical Engineering and Naval Architecture, University of Zagreb, Ul. Ivana Lučića 5, HR-10000 Zagreb, Croatia; suzana.jakovljevic@fsb.unizg.hr; 4 Department of Applied Chemistry, Faculty of Textile Technology, University of Zagreb, HR-10000 Zagreb, Croatia; iva.rezic@ttf.unizg.hr

**Keywords:** sol–gel process, ZnO, TiO_2_, modification, materials, anti-aging, color fastness, additive

## Abstract

The aim of this study was to prepare and characterize thin hybrid films on polyurethane-coated knitted fabrics and to achieve satisfactory color fastness to artificial light. Sol–gel-derived hybrid thin films were deposited via the dip-coating of 3-glycidoxypropiltrimethoxysilane. Titanium dioxide (TiO_2_) and zinc oxide (ZnO) nanopowders were added to compensate for the insufficient aging resistance, which manifests itself in low color fastness and is one of the most frequent complaints from manufacturers of coated marine fabrics (yachts, boats, etc.). The optimum processing conditions were determined by varying the concentration of precursors and auxiliaries, the mass concentration of TiO_2_ and ZnO nanopowders, the drawing speed, and the methods and process of fabric treatment. The hybrid films were also characterized using scanning electron microscopy and Fourier transform infrared spectroscopy with attenuated total internal reflection, while Spectraflash SF 300 investigated color fastness. After 300 h of exposure in a xenon chamber, the thin hybrid films showed good color fastness and good resistance to washing cycles. The sol–gel treatment proved to be a successful answer to the manufacturers’ need for the post-treatment of polyurethane-coated knitted fabrics against UV radiation for use in the marine sector (yachts, speedboats, etc.).

## 1. Introduction

Many maritime applications of polyurethane-coated knitted fabrics are now exposed to the effects of aging. These materials are increasingly used at sea, where the insufficient resistance to aging (sea water, UV radiation, moisture, etc.) of polyurethane-coated knitted fabrics for upholstery on yachts, speedboats, etc., is undesirable [1,2]. Manufacturers try to prevent aging and degradation effects by using various additives. However, some of them do not provide long-lasting and significant results. Customers consider abrasion resistance, color fastness, and mechanical properties to be very important. The main complaints concern color changes and loss of strength leading to small holes and similar defects. In some of the studies dealing with the aging of polyurethane and its use in the marine, the polyurethane samples were exposed directly to the sea over a longer period of time [1,3]. Those were not knitted fabrics coated with polyurethane, but panels used to insulate underwater cables and to recycle polyurethane waste. Most studies focused on the durability of polyurethanes under UV irradiation, with the most common technique for accelerating the aging of materials being to carry out tests in ovens that simulate UV radiation. The effect of aging is related to the mechanical properties of the material and is monitored by tensile tests. The studies were not concerned with textile samples, but with tiles [4]. The effect of aging on the structural changes in polyurethane/polyurea coatings on steel sheets was observed using four different types of aging: aging in the natural environment, accelerated ultraviolet aging, aging in a sodium chloride solution, and thermal aging. The results show that different aging conditions have a huge impact on appearance and pull-off strength. The coatings that age in a NaCl solution are more affected in terms of their adhesion [5]. Studies somewhat closer to our topic have investigated the changes in the properties of polyurethane-coated knitted fabrics for sportswear after exposure to sunlight. The effects of aging were related to mass, thickness, shrinkage, elongation, and breaking forces and tested [6]. Regardless of which polyurethane is used, with or without a textile component for maritime applications, it is necessary to provide it with additional protection against the effects of aging. As polyurethane is inherently sensitive to degradation (photo-oxidation, hydrolysis, and microbial degradation), its protection depends on the correct modification of its surface. Sol–gel coatings can prove very effective, regardless of whether they are applied to a textile or, for example, tiles [7,8]. Since this work deals with polyurethane-coated knitted fabrics, the sol–gel process is ideal for modification as it is versatile and offers the possibility of obtaining an inorganic phase at a low reaction temperature (usually below 100 °C), which is very important to avoid the degradation of sensitive compounds [9]. Textiles have acquired new properties through modification with nanosols, such as mechanical and thermal stability, as well as repellent and optical properties, with particular attention being paid to bioactive modifications [10,11]. Nanosols containing nanopowders such as TiO_2_ and ZnO have been reported to strongly absorb radiation [3,12,13,14]. In addition, it is reported that the preparation of nanosol coatings with TiO_2_ or ZnO particles smaller than 50 nm is advantageous because these coatings are colorless and transparent [12]. The application of the sol–gel process can be carried out with techniques normally used in the textile industry (e.g., the foularding process with subsequent thermal gelling), which is certainly an advantage. In addition, the sol–gel process can be carried out after all final textile processes, such as bleaching, dyeing, or mercerizing, which give the material its final appearance. In addition, the sol–gel process can be used to eliminate the defects of the material and provide it with an appearance and properties that satisfy the consumer (resistance to microorganisms, flame retardancy, antistatic resistance, etc.). The sol–gel modification of textiles was investigated on various textile substrates: polyacrylonitrile fabric, polyester, aramide non-woven textiles and technical p-aramide fabric, regenerate cellulose, cellulosic cotton (100%) and cotton/polyester (65/35%) fabrics, as well as on viscose fabrics [10,15,16]. Modifications included improved flame retardancy, resistance to surface wetting and to color change, UV absorption, and antimicrobial properties [12,15,17,18]. TiO_2_, ZnO nanoparticles are often added to hybrid coatings to improve their antibacterial and UV-resistant properties [10,18,19]. Other nanoparticles were also added, such as copper, silver, Fe_3_O_4_ [20,21,22,23]. As they are non-toxic and chemically stable against UV radiation at high temperatures, inorganic UV blockers such as TiO_2_, ZnO, and SiO_2_ are favored over organic UV blockers [24]. Therefore, in this work, ZnO and TiO_2_ nanoparticles were selected in order to improve the color fastness of polyurethane fabric. The most commonly used precursor for the sol–gel modification of textile materials is tetraethoxysilane (TEOS) [25,26,27,28]; other precursors include tetramethoxysilane (TMOS), methyltrimethoxysilane (MTMS), and 3-aminopropyltriethoxysilane (APTES) [10,29]. One of the more common organically modified precursors is 3-glycidyloxypropyltrimethoxysilane (GLYMO) [9,10,30,31] and its ethoxy derivative, 3-glycidyloxypropyltriethoxysilane (GLYEO) [32]. The advantage of organically modified precursors compared to TEOS is that they introduce functional organic groups into the sol–gel-derived coating, enabling properties such as surface wetting [10] and the better immobilization of auxiliary nanoparticles and nanopowders. In this work, GLYMO showed its potential as a precursor in that its three alkoxy groups contributed to the formation of a coating with a satisfactory textile character and it achieved a satisfactory color fastness to artificial light with ZnO or TiO_2_. The previous knowledge [9,33] about important parameters such as alkoxide concentration, water, alkoxide ratio, type of alkoxide groups, solvent used, temperature, the purity of chemicals used, stirring, etc., was used. In order to obtain reproducible hybrid materials, these parameters should not be changed. Alkoxides are very sensitive to humidity as this can affect the reaction of the sol–gel process. The sol–gel process seems very adaptable, an easy way to produce organic–inorganic hybrid materials, but a lot of carefully organized and conducted research, and practice is necessary to make it go smoothly. The relevant mechanism of the film obtained in this work is based on the sol–gel modification of coated textiles, i.e., polyester fabrics coated with polyurethane. Through the hydrolysis and condensation reactions of organically modified methoxysilane (GLYMO), the sol–gel process takes place with the formation of an uninterrupted three-dimensional silicon oxide network through two treatments. In one treatment, the hydrolysis reaction is catalyzed by acid; in the other treatment, there is no catalyst. The structure and morphology of the resulting molecular networks depends on the ratio of the rates of hydrolysis and the condensation reactions. The uncatalyzed hydrolysis of GLYMO was carried out with an excess of water and at room temperature, meaning that it proceeded very quickly and took less than an hour to complete the reaction. Furthermore, in neutral catalysis, the epoxide group is not hydrolyzed and crosslinking occurs only through hydrolysis and condensation of the alkoxide [34]. As with unsubstituted alkoxides, acidic catalysis causes the growth of a branched polysilsesquioxane chain with the limited formation of cyclic structures. The hydrolysis reaction of epoxy groups to diols is fast enough to cause condensation between the alcohol and silanol groups and the formation of intermolecular Si-O-Si bonds [34]. Therefore, the final structure and properties of the products of the sol–gel process can be specifically controlled by the reactivity parameter of the silicon alkoxide, the ratio of water to alkoxide, the concentration and purity of the reactants, the reaction temperature, the type of mixing, and the use of catalysts. In this work, parameters were chosen that allowed for treatments that ensured the retention of the textile character. In achieving the textile character, GLYMO proved to be an excellent precursor, leading to the formation of hybrid coatings that are soft and flexible to the touch, without distortion and brittleness. In our previous work, 2100 samples were coated to select the best treatment [9]. As the polyester fabric coated with polyurethane was not resistant to ultraviolet (UV) radiation with the manufacturer’s additives (based on the manufacturer’s complaint data), nanoparticles of ZnO and TiO_2_ were added. ZnO and TiO_2_ were added due to their ability to absorb ultraviolet and X-rays to achieve transparent coatings and effective UV protection for textiles.

This study focused on the manufacturers’ need for the post-treatment of polyurethane-coated knitted fabrics against UV radiation and our preliminary work, which indicated that hybrid coatings with nanoparticles can provide UV aging protection [35]. More specifically, the aim of this study was to prepare and characterize thin hybrid films on polyurethane-coated knitted fabrics and to achieve satisfactory color fastness to artificial light.

## 2. Materials and Methods

### 2.1. Preparation of Textile Materials

**Figure 1:**
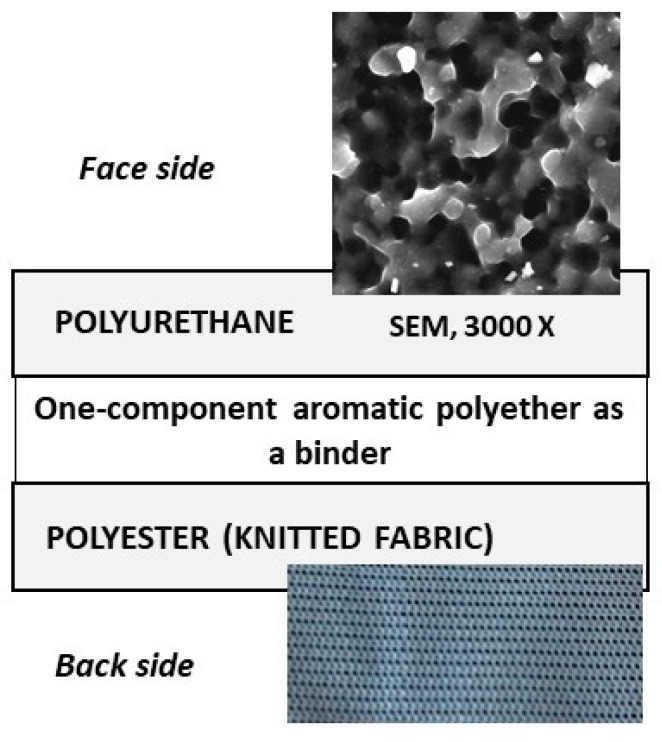
Cross-section of a knitted fabric coated with polyurethane.

Polyurethane-coated knitted fabrics donated by a local manufacturer for the marine program were used for the experiments. A sample was obtained that was modified by several treatments. Prior to the modifications, some basic properties of the textile were determined according to international standards. The composition of the sample was determined according to a method applicable to all polyurethane materials, to distinguish between polyester and polyether polyurethanes [36]. The sample consisted of a layer, i.e., a coated polymer (polyurethane) and a substrate, i.e., a carrier fabric (polyester) (Figure 1). The polyurethane was aromatic and between the layer and the substrate, a one-component aromatic polyether served as a binder. The textile material in question was treated with various additives during its manufacture, particularly to prevent aging, which are known only to the manufacturer. Mass per unit area was determined in accordance with ISO 2286-2 and was 175.9 g/m^2^ [37].

### 2.2. Preparation of Sols and Hybrid Materials

GLYMO (98%, Aldrich Chemicals, Sigma-Aldrich Chemicals, St. Louis, MO, USA) was hydrolyzed with hydrochloric acid (37%, Aldrich Chemicals) as an acidic catalyst, as well as with distilled water without the addition of a catalyst. Hydrochloric acid was chosen as the catalyst for hydrolysis because this volatile substance can be removed from the sol by evaporation before or during condensation. Nanopowders from Aldrich, titanium(IV) oxide (TiO_2_), with a particle size of less than 25 nm, and zinc oxide (ZnO) with a particle size of less than 50 nm, were used. Based on our previous studies, two treatments based on GLYMO were selected as particularly effective: treatment I (GLYMO hydrolyzed with pure water, GLYMO/water ratio of 1:3) and treatment II (GLYMO hydrolyzed with hydrochloric acid, GLYMO/water ratio of 1:1.5) [9]. In the first step of *treatment I*, the TiO_2_ nanopowder was thoroughly mixed with distilled water to produce a suspension. GLYMO was then added dropwise. The process was carried out with constant stirring for one hour. At the end, the mixture was poured into special Teflon pans for dip-coating. The same procedure was repeated for the ZnO nanopowder. In the first step of *treatment II*, distilled water, hydrochloric acid, and nanopowder were thoroughly mixed, and then GLYMO was added dropwise. The procedure was then continued as described above. For both treatments (I and II), GLYMO was hydrolyzed at 20 °C, the processing time was 1 h, and three mass concentrations of TiO_2_ and ZnO nanopowders were used separately. For each treatment, 20 samples measuring 5 × 5 cm were processed. The 5 × 5 cm samples were dip-coated at predetermined drawing speeds of 0.5 mm/s, 1 mm/s, 1.5 mm/s, and 2 mm/s. Dip-coating or chemical bath deposition (CBD) was chosen as a cost-effective method for the deposition of thin films in the nanometer range [38]. The modified samples were allowed to gel at room temperature for 24 h, and then dried at 100 °C for 1 h. No catalysts were used for the polymerization of the epoxy groups. The organic polymerization should not be complete, as this ensures less cross-linking and higher flexibility/softness in all organic domains. To prove the stability of the sol–gel coatings, i.e., to test the durability during washing, the samples were tested with the highest mass concentration of nanopowders after 10 washes according to EN ISO 6330 [39]. A schematic representation of the preparation of hybrid coatings can be found in Table 1.

Table 2 shows the codes of the samples, process conditions, and variations of the mass concentration of TiO_2_ and ZnO. 

### 2.3. Instrumental Methods

The surface structure and morphological properties of the coated textiles were investigated using the scanning electron microscope/energy-dispersive X-ray spectroscopy (SEM-EDS) TESCAN, VEGA TSS136LS (Los Angeles, CA, USA) with an operating voltage of 30 kV and a secondary electron detector. The FTIR spectra of the samples were recorded with the Bruker Vertex 70 instrument with attenuated total reflectance (ATR) in the range of 400–4000 cm^−1^. Thermogravimetric analysis (TGA) was performed using the Pyris 1 TGA from Perkin Elmer. Samples weighing approximately 17.0 mg were heated from room temperature to 1000 °C with a heating rate of 10 °C/min and a synthetic air flow of 30 cm^3^/min. The color fastness of the hybrid polyurethane-coated fabrics was determined according to EN ISO 105-B02: 2003, textiles—color fastness tests—color fastness to artificial light: Xenon arc lamp test: subjective (using the blue wool scales) and spectrophotometric [40]. The wool references ranged from 1 (very low color fastness) to 8 (very high fastness). The Spectraflash SF 300 (Datacolor, Dietlikon, Switzerland) reflectance spectrophotometer was used to measure the color parameters of the exposed samples.

## 3. Results

### 3.1. Results of Infrared Spectroscopy

**Figure 2:**
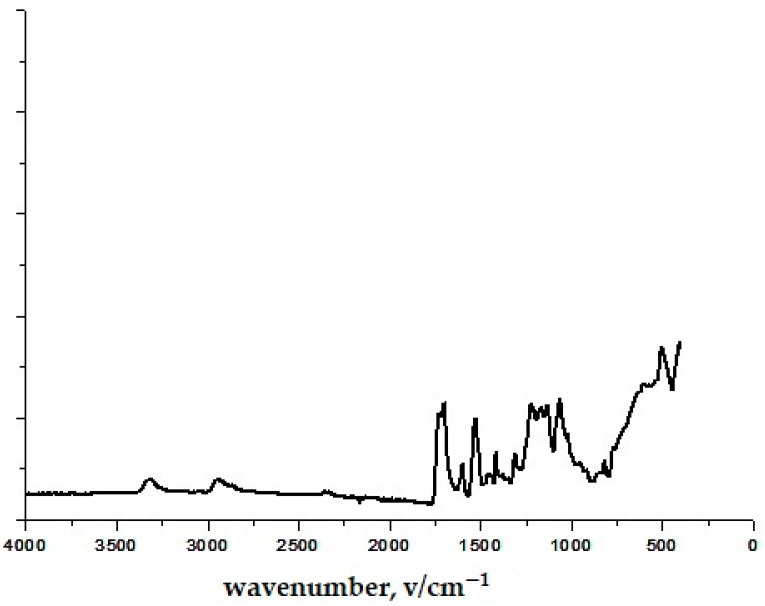
FTIR-ATR spectra of unmodified polyurethane-coated knitted fabrics.

**Figure 3:**
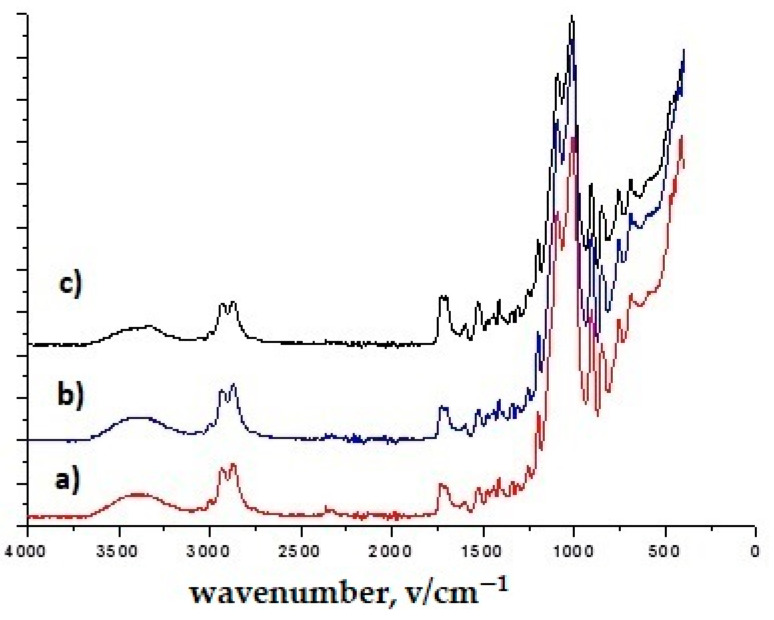
FTIR spectra of treatment I with (**a**) TiO_2_, 10 g/dm^3^ (1T); (**b**) TiO_2_, 50 g/dm^3^ (3T); (**c**) TiO_2_, 50 g/dm^3^, washed (3Tw).

**Figure 4:**
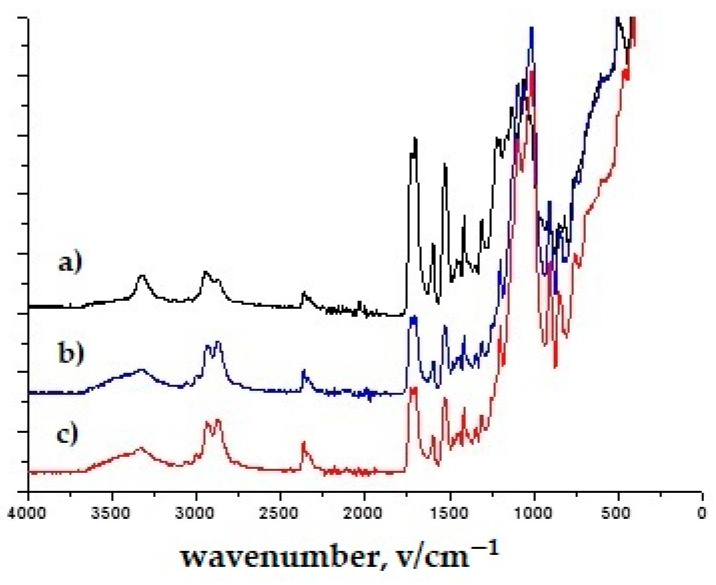
FTIR spectra of treatment II with (**a**) TiO_2_, 10 g/dm^3^ (4T); (**b**) TiO_2_, 50 g/dm^3^ (6T); (**c**) TiO_2_, 50 g/dm^3^, washed (6Tw).

**Figure 5:**
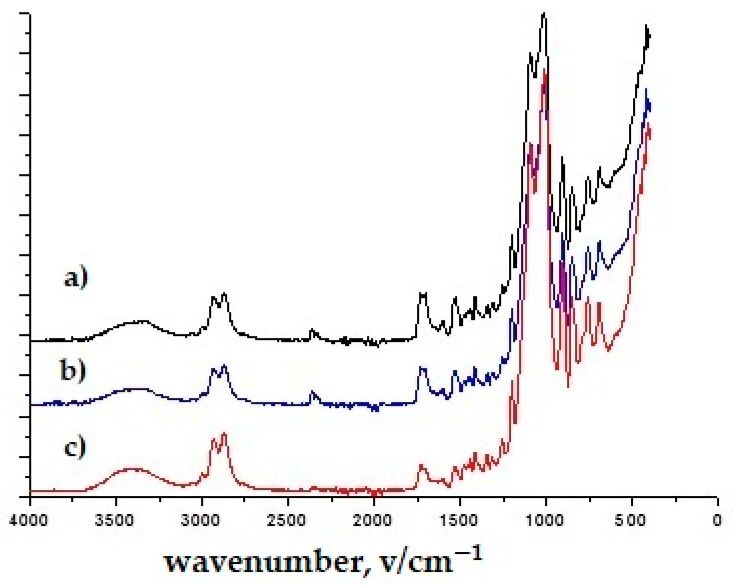
FTIR spectra of treatment I (**a**) ZnO, 10 g/dm^3^ (1Z); (**b**) ZnO, 50 g/dm^3^ (3Z); (**c**) ZnO, 50 g/dm^3^, washed (3Zw).

**Figure 6:**
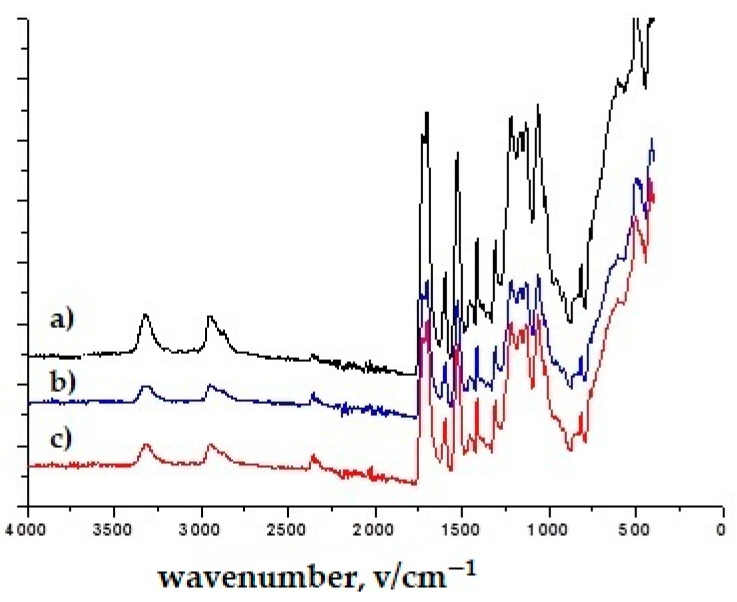
FTIR spectra of treatment II with (**a**) ZnO, 10 g/dm^3^ (4Z); (**b**) ZnO, 50 g/dm^3^ (6Z); (**c**) ZnO, 50 g/dm^3^, washed (6Zw).

The confirmatory evidence obtained through infrared spectroscopy was used for the composition, as shown in Figure 2, Figure 3, Figure 4, Figure 5 and Figure 6. The FTIR-ATR spectrum of polyurethane shown in Figure 2 has distinct bands at 3350 cm^−1^ belonging to N-H stretching; 2920 cm^−1^ corresponds to C-H stretching on the aromatic ring, 1750 cm^−1^ corresponds to C=O stretching; 1650 cm^−1^ corresponds to a deformation of the aromatic ring; 1531 cm^−1^ corresponds to N-H deformation; 1220 cm^−1^ corresponds to C-N stretching; 1103 cm^−1^ corresponds to C-O stretching [41,42]. By comparing this spectrum with previously known spectra, it was confirmed that it is polyurethane. In Figure 2, the C-O stretching and O-H deformation bands were identified at 1413–1472 cm^−1^. The bands in the 877 cm^−1^ region are associated with the benzene ring. A complementary band in the range of 712 cm^−1^ can be assigned to the angular deformation of (CH2)_n_. The characteristic bands were also identified and associated with the chemical structure of the polyester fiber [43]. In this way, the composition/structure of unmodified sample was initially elucidated using the FTIR-ATR method.

In the spectra of all TiO_2_ nanoparticles, the broad band between 400 and 800 cm^−1^ corresponds to the Ti–O–Ti network [44] (Figure 3 and Figure 4). Figure 3, Figure 4, Figure 5 and Figure 6 show the normalized FTIR spectra. The lower wavenumber regions of all spectra indicate the existence of nanoparticles. ZnO has been found to have bands at ~433 cm^−1^, ~780 cm^−1^, and ~1050 cm^−1^ (Figure 5 and Figure 6) [45]. In addition, the band at 1050 cm^−1^ overlaps with the band of the silicate network. In all FTIR spectra, the bands can be assigned between 1000 and 1200 cm^−1^ to the formation of Si-O-Si bonds due to the condensation of GLYMO. GLYMO reactive organic epoxide and hydrolysable inorganic metoxysilyl groups that hydrolyze in the presence of water and form reactive silanol groups that bond to inorganic substances (in our case nanoparticles) and to each other [45]. The FTIR spectra of treatment II contained distinct O-H bands (~3300 cm^−1^ and ~1640 cm^−1^; Figure 4 and Figure 6), in contrast to the FTIR spectra of treatment I. The bands can be assigned to silanol groups formed by hydrolysis of Si-O-CH_4_ groups. The small peak in the spectra of TiO_2_ at ~930 cm^−1^ confirms the condensation reaction between methoxysilyl groups (GLYMO) and the hydroxyl groups of the TiO_2_ surface [44]. Three different mass concentrations of nanopowders (TiO_2_, ZnO) had no significant effect on the infrared spectra, nor did the maintenance process (washed modified samples). The treated samples also showed the epoxy ring band at 904 cm^−1^, confirming the incomplete reaction of the organic component.

### 3.2. Thermal Properties

**Figure 7:**
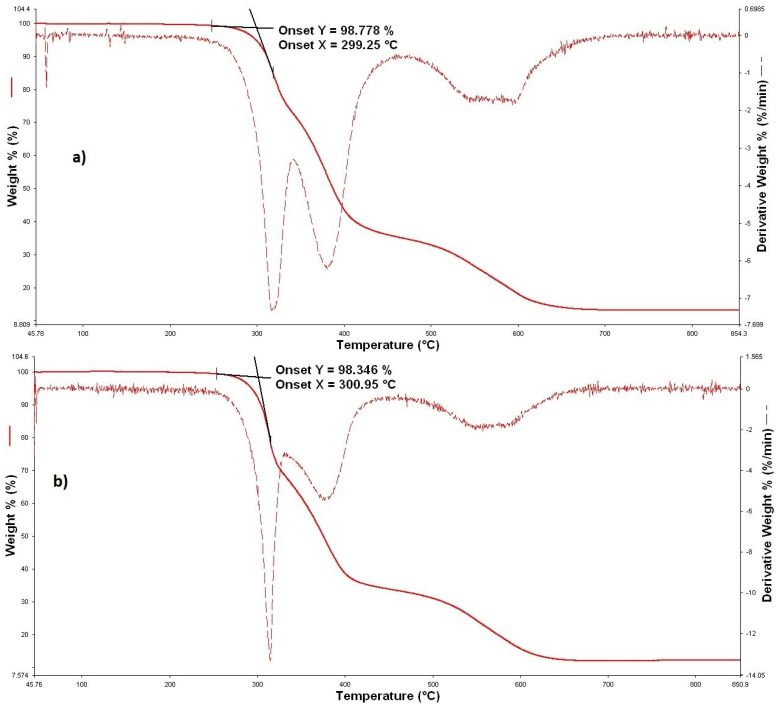
TGA curve of the degradation of (**a**) treatment I, TiO_2_, 50 g/dm^3^ (3T); (**b**) treatment I, TiO_2_, 50 g/dm^3^, washed (3Tw).

All parameters extracted from the TGA curves are summarized in Table 3. The TiO_2_ and ZnO treatments shifted the onset of degradation to slightly higher temperatures and showed a similar effect on the initial degradation process regardless of the hydrolysis conditions. It can be concluded that the morphology of the GLYMO hybrid coating with nanoparticles (TiO_2_ or ZnO) did not change significantly with the hydrolysis conditions. Only one treatment showed the second degradation process—treatment I, TiO_2_, 50 g dm^−3^ (Figure 7).

The final mass residues of the hybrid materials are higher (except treatment II, TiO_2_, 50 g/dm^3^—washed) than those of the unmodified sample. For all washed treatments, the final mass residue was lower, indicating that the maintenance process influenced and possibly partially removed the inorganic modification. As the hybrids did not burn completely at 1000 °C (~11–16%), it can also be assumed that the presence of the inorganic network led to the formation of less compact charcoal, ensuring easier combustion at higher temperatures.

### 3.3. Results of the Surface Structure and Morphological Characteristics

**Figure 8:**
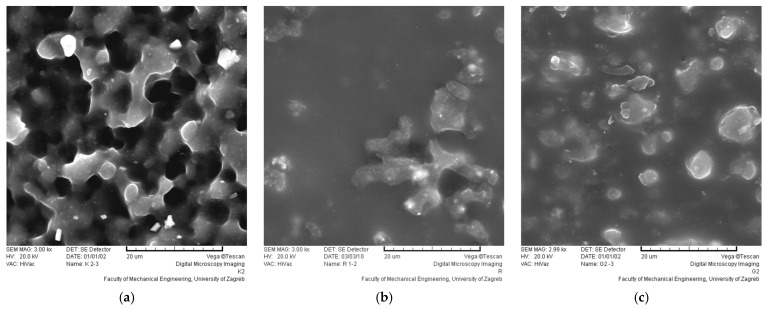
Surface of (**a**) unmodified samples and samples modified by (**b**) treatment I, (without nanopowder); (**c**) treatment II (without nanopowder); size bar is 20 μm in all images.

**Figure 9:**
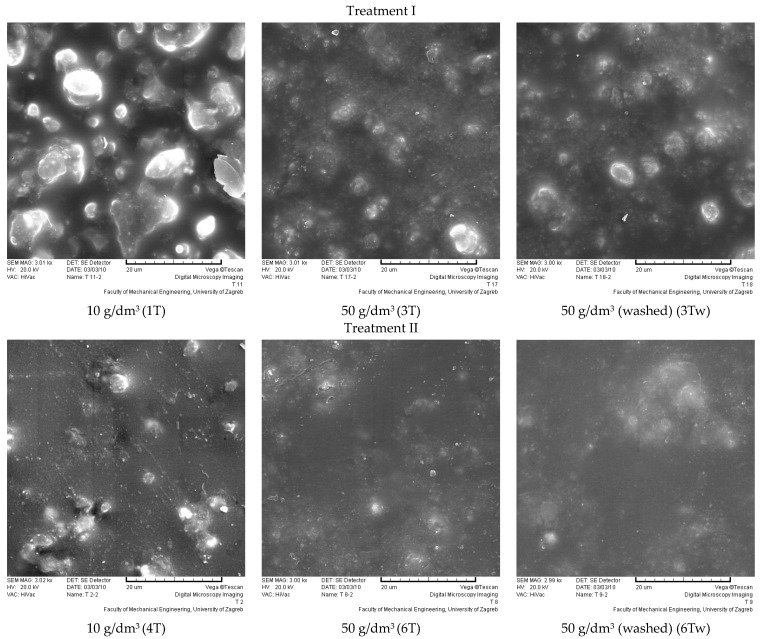
Surface of samples modified with TiO_2_ nanopowder; size bar is 20 μm in all images.

**Figure 10:**
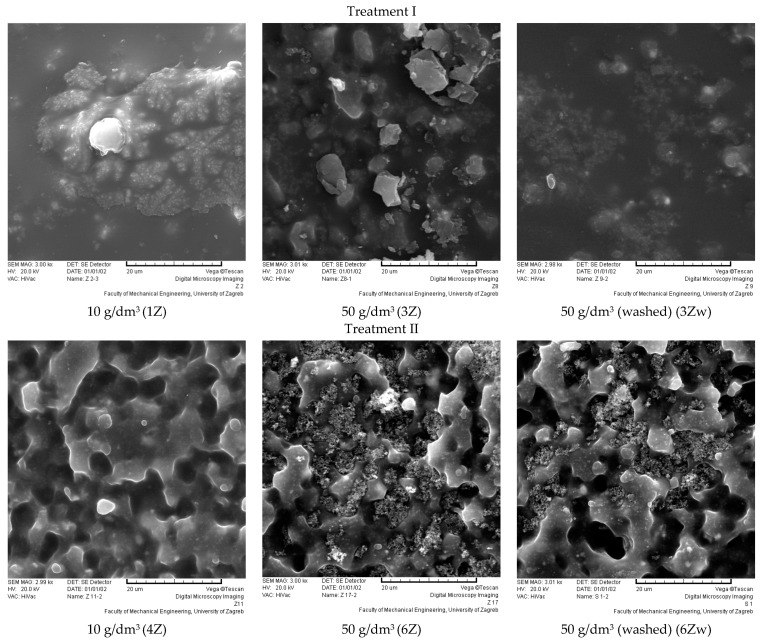
Surface of samples modified with ZnO nanopowder; size bar is 20 μm in all images.

The SEM images of the coating surfaces (Figure 8, Figure 9 and Figure 10) show the modification of the polyurethane by the sol–gel process, which was applied at a drawing speed of 1 mm/s via immersion. The SEM images of the coating surface were used to see how successful the modifications were in “capturing” manufacturer additives in the polyurethane coating, as these are often removed during maintenance and by various aging effects. It is hypothesized that unmodified polyurethane-coated samples will show the additives through the pores. The additives are irregularly circular and unevenly distributed in the sample. On all SEM images, they can be recognized as lighter in color and irregularly circular. They are present after the sol–gel modification.

The polyurethane-coated surface showed different behavior after modification with ZnO and TiO_2_, as well as with treatments I and II. The surface of the samples modified with treatments I and II (TiO_2_) is completely covered with hybrid thin films, forming an organic–inorganic composite coating. The nanopowder of TiO_2_ is present in the sol–sol–gel coating, with all TiO_2_ coatings showing an irregular distribution with the sporadic agglomeration of nanopowder. There was no visually significant difference after care.

For the ZnO sol–gel coatings, the difference between treatment I and II is clearly visible, even with different amounts of nanopowder. Treatment II (ZnO) has an open structure, typical of an unmodified polyurethane, with nanopowder unevenly distributed between the pores. With a higher amount of nanopowder, the pores are fuller. Care (washing) had no visual effect on the sol–gel coating and the amount of nanopowder present. It can be assumed that all modifications are well-suited to “capture” the manufacturer’s additives in the polyurethane coating.

The results of the microanalysis of the chemical elements on the surface of the sol–gel coatings are shown in Table 4. The 25 × 25 μm^2^ area in the center of each sample was used to collect the EDS data.

Unmodified samples contain carbon (C), oxygen (O), aluminum (Al), and titanium (Ti). The presence of Ti could be due to the masterbatch process during production, while Al could be the result of the coating on the sputter coating prior to SEM-EDS analysis or overlap with other chemical elements. The difference between treatments I and II lies in the amount of Si (treatment I 3.63 wt%; treatment II 17.50 wt%); treatment I contains Al and treatment II contains Ti. The microanalysis of ZnO shows the presence of C, O, Si, Ti, and Zn. After washing, Cl, Al, and Zn are also present. After washing, the amount of Si decreased and Zn is no longer present. The thermal analysis for ZnO (treatment I) does not show a very clear decrease in the mass residue after washing. It can be assumed that washing removed the ZnO from the surface of the coating, but that some ZnO remains in the pores of the fabric, below the detection limit of the EDS. Treatment II has a higher Si content and both treatments have a satisfactory wash resistance (low loss of Si). The low loss of Ti is consistent with the results of the thermal analysis. A microanalysis of the chemical elements was performed to verify the presence of Si. The result of the SEM analysis suggests that the polyurethane-coated fabric is covered with the hybrid film.

### 3.4. Results of Color Fastness

**Figure 11:**
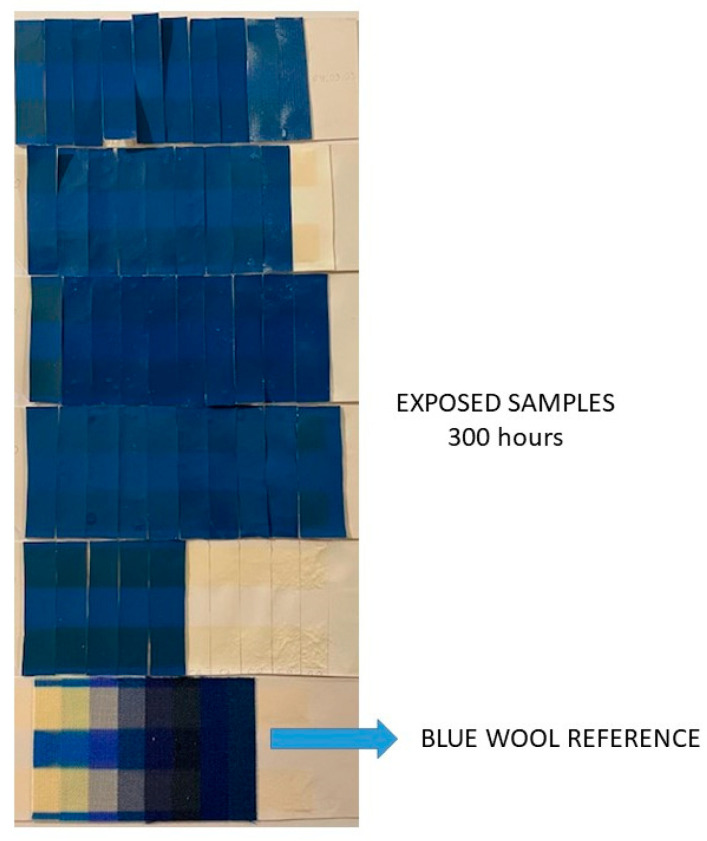
Example of light fastness testing of polyurethane-coated knitted fabric and blue wool standards.

The samples were exposed to artificial light under the prescribed conditions according to ISO 105-B02, together with a series of blue wool references (Figure 11). The blue wool references are identified by a numerical designation from 1 to 8 and were dyed with the dyes listed in the standard. Color fastness was assessed by comparing the color change in the test samples with that of the reference used. Exposure to light was monitored until the change in the test sample corresponded to gray scale 4. At this point, the number of blue wool references with a similar change in color was noted. The final evaluation, in the form of a numerical classification, was based on the contrast between the exposed and unexposed test sample, which corresponds to the gray scale value 4. In this work, the samples were exposed to the xenon arc lamp test for 300 h, which corresponds to the change in blue wool reference 7 (8 very high fastness), gray scale grade 4, for almost all samples. The term “color change” includes the change in the hue, chroma, lightness or any other characteristic of the color.

Table 5 and Table 6 show the color change and coloring according to the gray scale and ΔE, CIELAB. ΔE is a single number that indicates the distance between two colors, i.e., how far the thin hybrid coatings were from the unmodified coatings after being exposed to the xenon arc lamp test for 300 h.

The color fastness of the ZnO thin-film hybrid coating for the modified samples showed good fastness in both treatments (I and II), even after washing (grades 6 and 7). The samples modified with GLYMO and ZnO (treatment I) and higher mass concentrations (20 g/dm^3^, 50 g/dm^3^) resulted in a higher degree of color fastness. It is obvious that the use of ZnO as an inorganic UV absorber contributed to the good results. Table 4 shows that there was no significant effect of drawing speed on the results, but the mass concentration had an influence, especially in treatment I (20 g/dm^3^, 50 g/dm^3^). The highest value of ΔE was recorded for the unmodified sample with a manufacturer’s additive (6.79), while the thin ZnO hybrid layers had values ranging from 1.86 to 4.34.

The color fastness of the TiO_2_ thin-film hybrid coating for the modified samples showed good resistance in both treatments (I and II), even after washing (grades 6 and 7). The samples modified with GLYMO and TiO_2_ nanopowder showed a higher degree of color fastness at a higher mass concentration (50 g/dm^3^). There was almost no difference in color fastness for the thin TiO_2_ hybrid coatings. It is obvious that the use of TiO_2_ as an inorganic UV absorber contributed to the good results, as did the use of ZnO. Table 6 shows that the drawing speed had no significant effect on the color fastness results, but the mass concentration had an influence, especially for treatment I (20 g/dm^3^, 50 g/dm^3^) and treatment II (50 g/dm^3^).

The values of ΔE for thin TiO_2_ hybrid coatings ranged from 1.51 to 4.41, while the value of ΔE for the unmodified sample was 6.79. TiO_2_ or ZnO completely absorb the light at a higher energy than their band gap energy (e.g., Egap/TiO_2_ = 3.0 eV; Egap/ZnO = 3.2 eV). This band gap energy strongly depends on the type and degree of the crystallinity of the inorganic coatings [46]. In this work, TiO_2_ or ZnO provided optimal protection, i.e., good color fastness. To prevent the aging effects of polyurethane-coated fabrics, in particular the reduction in color fastness, inorganic UV absorbers, such as TiO_2_ and ZnO, were the obvious solution. However, UV-protective coatings, which should absorb practically all UV light, inorganic and organic UV absorbers are required, which are embedded in the structure (e.g., phenyl acrylates), again by a sol–gel process.

Numerous available literature sources emphasize the advantage of using one of the precursors with TiO_2_ and ZnO against UV radiation in terms of the resistance of the coating, regardless of the fabrics, as a large number of papers refer specifically to cotton, polyester, polyamide, polyethylene-coated polyester fabrics, and others [47,48]. Interestingly, the p-aramid fabric coated with GYLMO, ZnO, and bisphenol A exposed to UV radiation retained its tensile strength and protective properties, while the untreated fabric lost 80% of its tensile strength. Similarly, the authors prepared coatings with TiO_2_ instead of ZnO, with the results showing the same properties as coatings prepared with ZnO [10]. Numerous studies have shown that ZnO and TiO_2_ are ideal for coatings when it comes to absorbing negative energy (the absorption of ultraviolet and X-rays), both on textiles and in other industries [49,50,51].

## 4. Conclusions

Hybrid thin films were produced on polyurethane-coated fabrics using the sol–gel process, with the addition of TiO_2_ and ZnO. The hybrid thin films provided good color fastness after 300 h of exposure in a xenon chamber and were helpful in meeting manufacturers’ requirements quickly. Treatments based on GLYMO and TiO_2_ and ZnO nanopowders are suitable for the modification of the investigated textiles. Such modifications of the textile surface via sol–gel processes could lead to new properties of the polyurethane-coated fabrics, particularly a higher aging resistance. Thin TiO_2_ and ZnO hybrid coatings could also be produced on other textiles; for example, on woven or knitted fabrics and yarns. The samples coated with two GLYMO-based treatments remained undistorted after gelling; their coating had more luster and was almost imperceptible to the touch.

## Figures and Tables

**Table 1:** Schematic representation of the preparation of hybrid coatings.

Treatments					
**↙**			**↘**		
Treatment I GLYMO hydrolized with pure water GLYMO:water ratio of 1:3			Treatment II GLYMO hydrolized with catalyst (water, ethanol 1.6 mol/L, hydrochloric acid 0.1 mol/L) GLYMO:water ratio of 1:1.5		
**↙** + TiO_2_ (10 g/dm^3^)	**↓** + TiO_2_ (20 g/dm^3^)	**↘** + TiO_2_ (50 g/dm^3^)	**↙** + TiO_2_ (10 g/dm^3^)	**↓** + TiO_2_ (20 g/dm^3^)	**↘** + TiO_2_ (50 g/dm^3^)
**↓** continuous magnetic stirring 20 ± 2 °C processing time 1 h					
**↓** samples 5 × 5 cm in size → dip-coated Teflon pans drawing speed of 0.5 mm/s, 1 mm/s, 1.5 mm/s, 2 mm/s					
**↓** modified samples left to gel at room temperature for 24 h dried at 100 °C for 1 h					
**↓** FITR-ATR SEM-EDS TGA Xenon arc lamp test (EN ISO 105-B02) → colour fastness (blue wool scales and Spectraflash SF 300) modified samples with the highest mass concentration → durability during washing (EN ISO 6330)					

The same procedure was repeated for the ZnO.

**Table 2:** Codes of the samples, process conditions, and variations of the mass concentration of TiO_2_ and ZnO.

Code	Mass Concentration	Treatment
0	/	unmodified
I	/	Treatment I
II	/	Treatment II
1T	10 g/dm^3^	Treatment I + TiO_2_
2T	20 g/dm^3^	Treatment I + TiO_2_
3T	50 g/dm^3^	Treatment I + TiO_2_
3Tw	50 g/dm^3^	Treatment I + TiO_2_—washed
4T	10 g/dm^3^	Treatment II + TiO_2_
5T	20 g/dm^3^	Treatment II + TiO_2_
6T	50 g/dm^3^	Treatment II + TiO_2_
6Tw	50 g/dm^3^	Treatment II + TiO_2_—washed
1Z	10 g/dm^3^	Treatment I + ZnO
2Z	20 g/dm^3^	Treatment I + ZnO
3Z	50 g/dm^3^	Treatment I + ZnO
3Zw	50 g/dm^3^	Treatment I + ZnO—washed
4Z	10 g/dm^3^	Treatment II + ZnO
5Z	20 g/dm^3^	Treatment II + ZnO
6Z	50 g/dm^3^	Treatment II + ZnO
6Zw	50 g/dm^3^	Treatment II + ZnO—washed

**Table 3:** Process onset temperature *t_onset_*, temperatures of two degradation maxima, *t_max,_*_1_ and *t_max_,*_2_, and residual mass content, as determined by TGA.

Treatment			*t_onset_* (°C)	*t_max_*_,1_ (°C)	*t_max_*_,2_ (°C)	Residue (%)
Unmodified			296.45	310	/	11
TiO_2_	Treatment I	50 g/dm^3^ (3T)	302.83	318	381	14
		50 g/dm^3^—washed (3Tw)	301.96	315	/	12
	Treatment II	50 g/dm^3^ (6T)	305.00	317	/	12
		50 g/dm^3^—washed (6Tw)	306.47	319	/	11
ZnO	Treatment I	50 g/dm^3^ (3Z)	300.09	330	/	15
		50 g/dm^3^—washed (3Zw)	306.25	328	/	14
	Treatment II	50 g/dm^3^ (6Z)	295.64	319	/	16
		50 g/dm^3^—washed (6Zw)	299.05	319	/	14

**Table 4:** Number ratios of elements on the surface of coatings as determined by EDS.

Sample		Element	(Weight%)	(Atomic%)
Unmodified (0)		C	59.11	68.15
		O	33.04	28.60
		Al	4.39	2.25
		Ti	3.46	1.00
Treatment I		C	55.35	64.97
		O	35.18	31.00
		Al	2.15	1.12
		Si	3.63	1.82
		Ti	3.70	1.09
Treatment II		C	47.30	58.74
		O	33.65	31.37
		Si	17.50	9.29
		Cl	1.05	0.44
		Ti	0.51	0.16
ZnO Treatment I	50 g/dm^3^ (3Z)	C	45.21	56.85
		O	41.92	39.58
		Si	1.06	0.57
		Ti	3.26	1.03
		Zn	8.56	1.98
	50 g/dm^3^ washed (3Zw)	C	52.73	61.62
		O	39.01	34.22
		Al	7.43	3.71
		Si	0.35	0.21
		Cl	0.34	0.18
		Ti	0.13	0.05
		Zn	0	0
TiO_2_ Treatment II	50 g/dm^3^ (6T)	C	43.52	55.35
		O	36.78	35.12
		Si	14.43	7.85
		Ti	5.27	1.68
	50 g/dm^3^ washed (6Tw)	C	44.05	54.96
		O	39.73	37.22
		Si	12.24	6.53
		Cl	0.48	0.20
		Ti	3.50	1.09

**Table 5:** The results of color fastness for ZnO thin hybrid coatings.

Treatment/Mass Concentration of ZnO, Drawing Speed		Change in Color			
		Treatment I		Treatment II	
		Gray Scale	Δ*E*	Gray Scale	Δ*E*
10 g/dm^3^	0.5 mm/s	6	3.16	6	3.76
	1 mm/s	6	3.61	6	4.22
	1.5 mm/s	6	3.82	6	4.34
	2 mm/s	6	3.66	6	4.13
	1 mm/s, washed	6	3.90	6	4.00
20 g/dm^3^	1 mm/s	7	3.05	6	4.17
	1 mm/s, washed	7	2.74	6	3.72
50 g/dm^3^	1 mm/s	7	3.30	6	3.79
	1 mm/s, washed	7	1.86	6	3.38
Unmodified (producer additive): 4–5 (gray scale); 6.79 (ΔE)					

Where: ΔE (CIELAB); gray scale for changes in color and staining.

**Table 6:** The results of TiO_2_ thin hybrid coating color fastness.

Treatment/Mass Concentration of ZnO, Drawing Speed		Change in Color			
		Treatment I		Treatment II	
		Gray Scale	Δ*E*	Gray Scale	Δ*E*
10 g/dm^3^	0.5 mm/s	6	3.70	6	3.67
	1 mm/s	6	3.68	6	3.95
	1.5 mm/s	6	3.66	6	4.31
	2 mm/s	6	3.81	6	4.41
	1 mm/s, washed	6	3.41	6	3.76
20 g/dm^3^	1 mm/s	7	3.40	6	4.09
	1 mm/s, washed	7	2.92	6	3.38
50 g/dm^3^	1 mm/s	7	3.01	7	3.56
	1 mm/s, washed	7	1.51	7	2.25
Unmodified (producer additive): 4–5 (gray scale); 6.79 (ΔE)					

Where: ΔE (CIELAB); gray scale for change in color and staining.

## Data Availability

The original contributions presented in the study are included in the article; further inquiries can be directed to the corresponding author.
